# Conversion to Hip Arthroplasty After Internal Fixation Failure in an Intertrochanteric Femoral Fracture: A Case Report

**DOI:** 10.7759/cureus.90112

**Published:** 2025-08-14

**Authors:** Shiro Fukuoka, Tomoo Inoue, Motoki Takahashi, Keisuke Kawasaki, Toshifumi Ozaki

**Affiliations:** 1 Department of Orthopedics, Kagawa Prefectural Central Hospital, Takamatsu, JPN; 2 Department of Orthopedic Surgery, Kagawa Prefectural Central Hospital, Takamatsu, JPN; 3 Department of Orthopedic Surgery, Okayama University Graduate School of Medicine, Dentistry and Pharmaceutical Science, Okayama, JPN

**Keywords:** arthroplasty, coronal shear fracture, double jaws sign, fixation failure, intertrochanteric femoral fracture

## Abstract

Intertrochanteric femoral fractures are mainly managed by internal fixation. However, failures such as over-telescoping, cut-out, nonunion, or implant failure can occur, especially in osteoporotic elderly patients. We report the case of a patient in whom we performed artificial hip replacement surgery after fixation failure following internal fixation of an intertrochanteric femoral fracture. We report the case of an 85-year-old woman who sustained a left intertrochanteric femoral fracture treated with a dynamic hip screw (DHS). One week postoperatively, radiographs revealed over-telescoping of the lag screw. The patient did not complain of pain, but she underwent conversion to cemented bipolar hemiarthroplasty under general anesthesia. One possible cause of over-telescoping of the lag screw after surgery was that the longitudinal fracture line in the calcar of the proximal bone fragment, as seen in the initial CT image, may have extended horizontally at the neck level. During surgery, a fracture at the same site caused the anterior medial fragment to fail, resulting in a coronal shear fracture and fixation failure. When a longitudinal fracture line is observed in the calcar of the proximal fragment, it is necessary to keep in mind that it may extend horizontally at the neck level.

## Introduction

Intertrochanteric femoral fractures are becoming increasingly common as the population ages [1]. Internal fixation using devices such as sliding hip screws or intramedullary nails is the first choice for treating intertrochanteric femoral fractures. However, the failure rate of fixation varies widely (reaching up to approximately 56% in unstable or reverse-oblique fractures) [2]. Risk factors for cut-out failure in a different generation of intramedullary nails have been reported, including fracture pattern, quality of reduction, tip-apex distance, and lag screw position [3]. In this report, we diagnosed a stable intertrochanteric femoral fracture and chose DHS fixation, but internal fixation failure occurred early after surgery. The fracture line from the calcar extended horizontally at the neck level, and an anterior medial bone fragment was generated at the same level after surgery. One possible cause of the fixation failure may be related to the type of fracture.

## Case presentation

**Figure 1:**
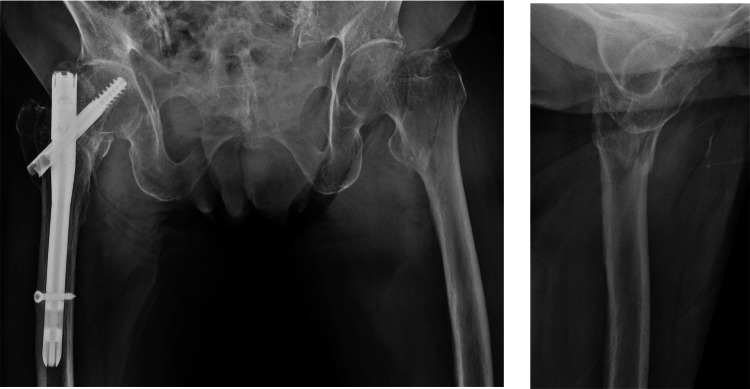
Pre-operative X-ray images of the hip. A fracture line was visible in the intertrochanteric region of the left proximal femur, with minimal displacement.

**Figure 2:**
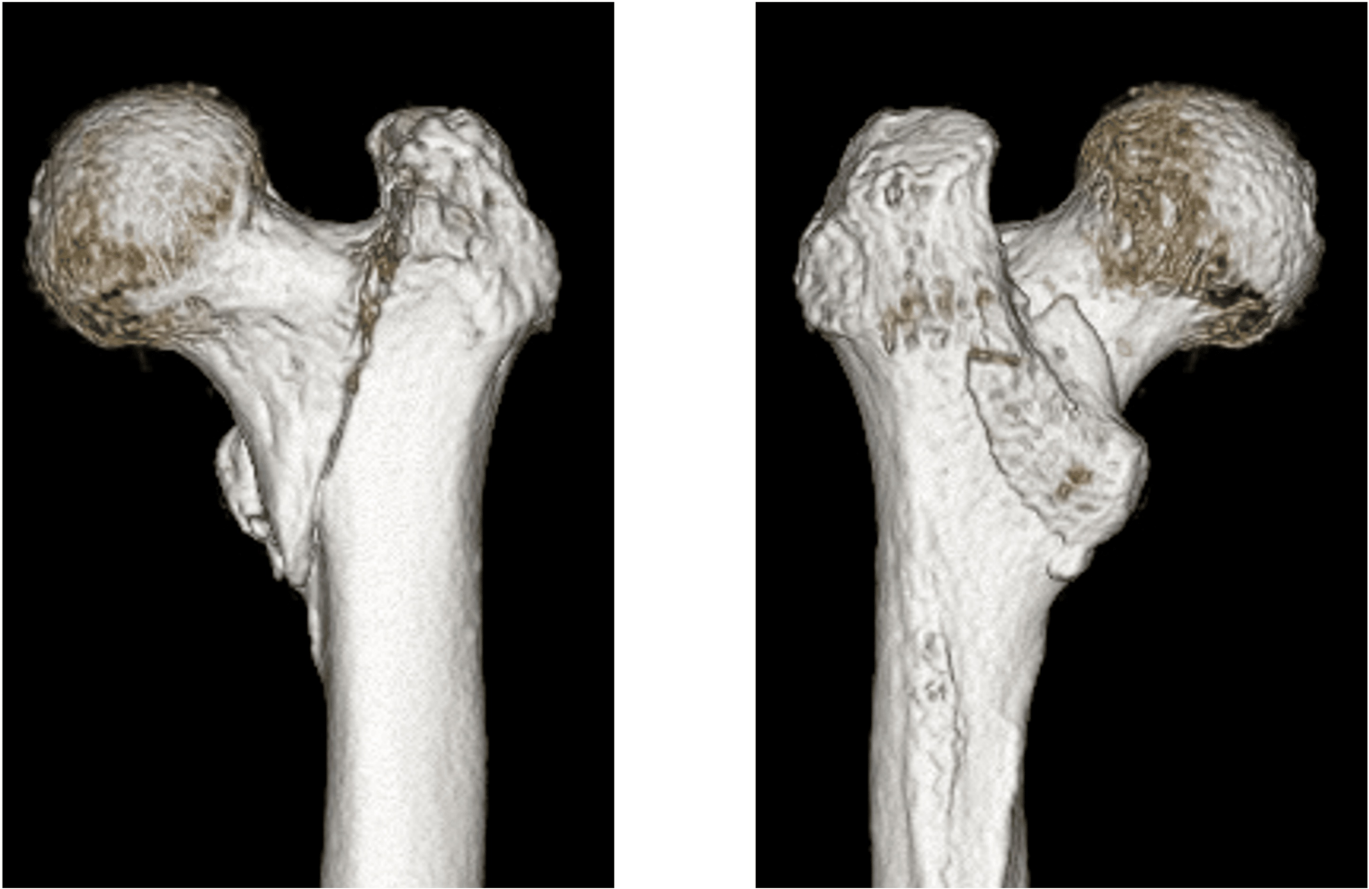
Pre-operative 3D computed tomography (CT) images of left hip. A main fracture line was observed at the intertrochanteric line, and a bone fragment including the lesser trochanter was observed posterior medially.

**Figure 3:**
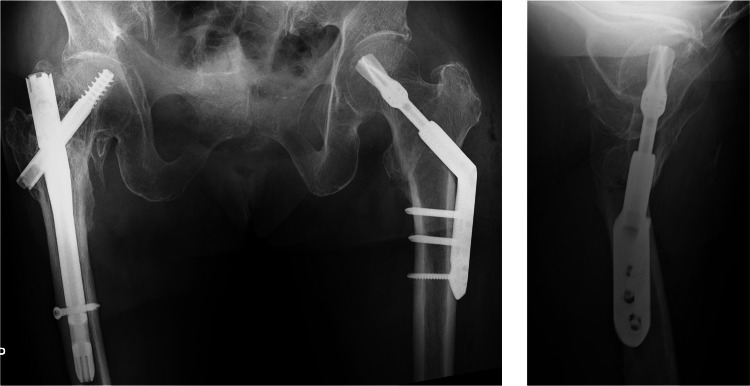
Postoperative X-ray images of the hip. Internal fixation by DHS was performed.

An 85-year-old woman with a history of right intertrochanteric femoral fracture sustained a left intertrochanteric femoral fracture (AO/OTA 31A1.3, Jensen classification type Ⅱ) from slipping at home (Figures 1, 2). Treated with DHS (Figure 3), postoperative reduction was judged to be good according to Baumgaertner criteria. She was pain-free and ambulatory with a walker initially.

**Figure 4:**
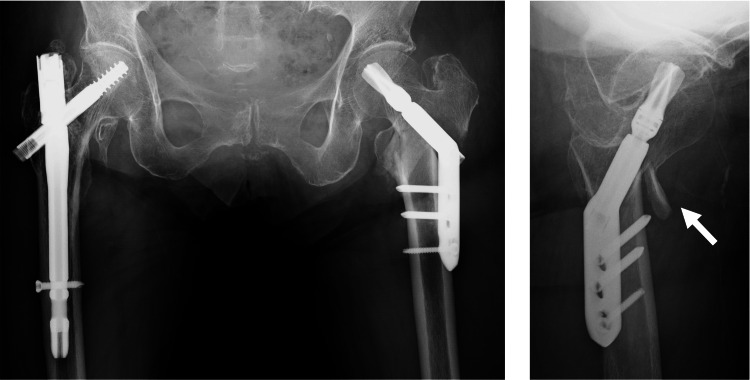
X-ray images of the hip one week postoperatively. Over-telescoping of the lag screw was observed, and the anterior medial bone fragment was observed in the lateral image (white arrow).

**Figure 5:**
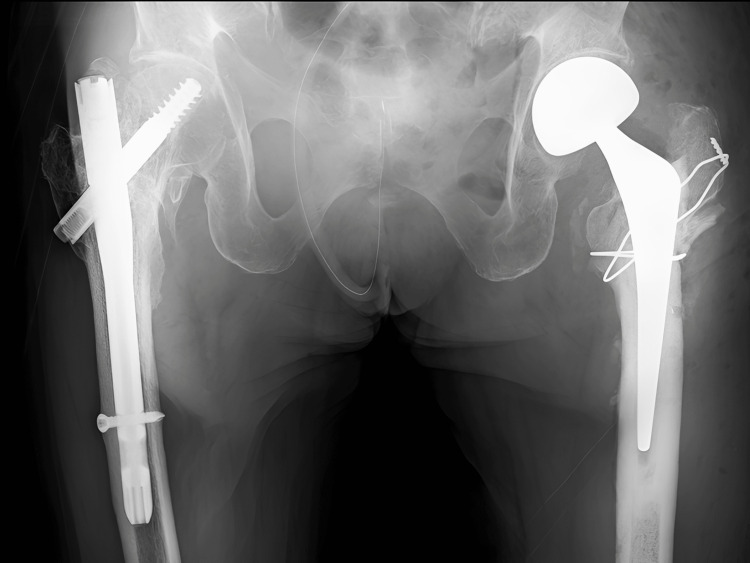
X-ray images after reoperation (cemented bipolar hemiarthroplasty). The unstable greater trochanter was stabilized by wiring.

However, one week postoperatively, radiographs revealed over-telescoping of the lag screw (Figure 4). The patient did not complain of pain, but given the poor bone quality and failure of fixation, conversion to arthroplasty was chosen (Figure 5).

At surgery, the DHS was removed, and due to instability in the greater trochanter during surgery, wire fixation was necessary. A cemented bipolar hemiarthroplasty stem was implanted. The surgery duration was 225 minutes, with a blood loss of 500 mL. No fractures were observed during the surgery. Postoperatively, she started assisted weight-bearing on Day 1 and regained her ability to walk independently with the help of a walker.

## Discussion

In the AO/OTA classification, 31A1 fractures are classified as stable, and even when fixed with a sliding hip screw, postoperative failure of fixation is unlikely to occur. Clinical results are the same as those of cephalomedullary nails, and because it is relatively economical, sliding hip screws are generally recommended [4].

**Figure 6:**
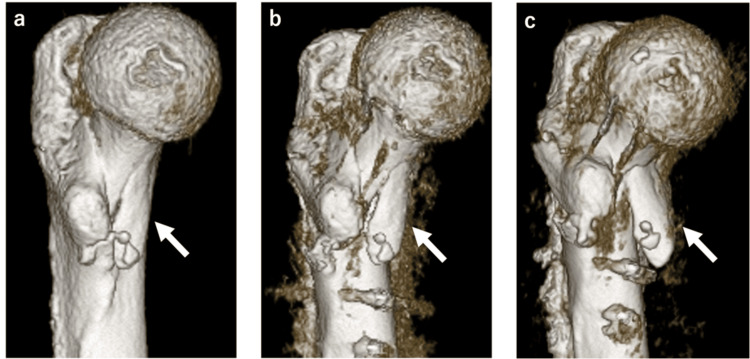
Temporal changes in 3DCT from preoperative to postoperative fixation failure. 3DCT images taken pre-operatively (a), the day postoperatively (b), and one week postoperatively (c). The anterior medial fragment was displaced (white arrow).

**Figure 7:**
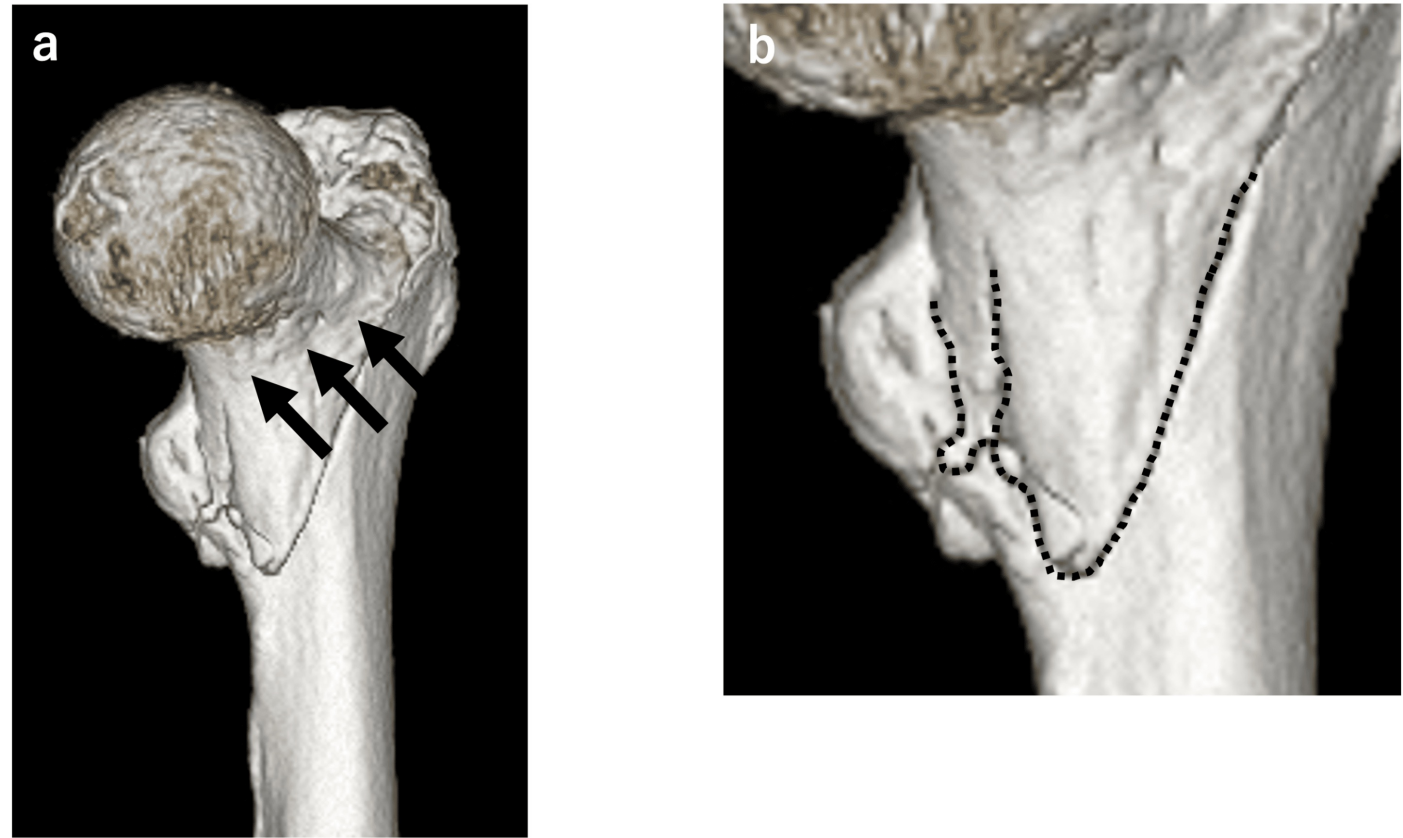
Pre-operative 3D CT images. (a) The fracture line from calca to the proximal direction appears to extend horizontally at the level of the neck (black arrows). (b) "Butt" sign (black dotted line).

As for causes of internal fixation failure in this case, when looking at the temporal changes in pre-operative and postoperative 3D CT images (Figure 6), it is thought that the fracture line in the calcar of the proximal bone fragment, which is a longitudinal fracture in the proximal direction, extended horizontally at the neck level (Figure 7a).

It is speculated that the failure of the anterior medial fragment postoperatively may have contributed to the development of the basicervical fracture at the coronal plane.

Yamakawa et al. reported coronal shear fracture of the femoral neck (CSFF) as a fracture type in which the fracture line resembles an inverted J curve, and the anterior cortical fracture line is located close to the femoral head [5]; a similar fracture line was observed in this case. In other words, the initial diagnosis was an intertrochanteric femoral fracture, but it also had elements of CSFF. 

One of the problems with basicervical fracture is poor postoperative results after internal fixation. This is due to the lack of soft tissue attachment to the proximal fragment and the small area of the fracture surface, which allows the proximal fragment to be easily rotated and shifted [6,7]. Intramedullary nails and DHS are often the treatment of choice [8-10], but in elderly patients, arthroplasty may be the treatment of choice for the initial surgery [11].

To the best of our knowledge, there have been no reports of cases in which CSFF occurred after internal fixation of an intertrochanteric fracture.

In this case, the proximal bone fragment is divided into two parts at the calcar, and because it resembles a buttock, it has been named the “Butt sign” (Figure 7b). If this sign is recognized, it is necessary to keep in mind the possibility that the fracture line extends to the neck level from the calcar of the proximal bone fragment. As a surgical option, treatment like that for coronal shear fractures is necessary, and in the case of elderly patients, the selection of an arthroplasty must also be considered from the outset.

## Conclusions

When a longitudinal fracture toward the proximal direction at the calcar is observed, it is necessary to accurately evaluate the fracture type, taking into consideration the possibility that the fracture line may extend to the neck level. In this case, considering due to the advanced age of the patient, osteoporosis, and the presence of CSFF, the fracture site was highly unstable, and internal fixation could have caused internal fixation failure. It may have been more appropriate to opt for arthroplasty during the initial surgery. Further accumulation of similar cases would aid in refining guidelines for optimal management.
